# Evolutionary conservation and divergence of the transcriptional regulation of bivalve shell secretion across life-history stages

**DOI:** 10.1098/rsos.221022

**Published:** 2022-12-21

**Authors:** Alessandro Cavallo, Melody S. Clark, Lloyd S. Peck, Elizabeth M. Harper, Victoria A. Sleight

**Affiliations:** ^1^ Biodiversity, Evolution and Adaptation Team, British Antarctic Survey, Cambridge CB3 0ET, UK; ^2^ Department of Earth Sciences, University of Cambridge, Cambridge CB2 1TN, UK; ^3^ Department of Zoology, University of Cambridge, Cambridge CB2 1TN, UK; ^4^ School of Biological Sciences, University of Aberdeen, Aberdeen AB24 3FX, UK

**Keywords:** shell development, evo devo, mollusc, biomineralization

## Abstract

Adult molluscs produce shells with diverse morphologies and ornamentations, different colour patterns and microstructures. The larval shell, however, is a phenotypically more conserved structure. How do developmental and evolutionary processes generate varying diversity at different life-history stages within a species? Using live imaging, histology, scanning electron microscopy and transcriptomic profiling, we have described shell development in a heteroconchian bivalve, the Antarctic clam, *Laternula elliptica,* and compared it to adult shell secretion processes in the same species*.* Adult downstream shell genes, such as those encoding extracellular matrix proteins and biomineralization enzymes, were largely not expressed during shell development. Instead, a development-specific downstream gene repertoire was expressed. Upstream regulatory genes such as transcription factors and signalling molecules were largely conserved between developmental and adult shell secretion. Comparing heteroconchian data with recently reported pteriomorphian larval shell development data suggests that, despite being phenotypically more conserved, the downstream effectors constituting the larval shell ‘tool-kit’ may be as diverse as that of adults. Overall, our new data suggest that a larval shell formed using development-specific downstream effector genes is a conserved and ancestral feature of the bivalve lineage, and possibly more broadly across the molluscs.

## 1. Introduction

Molluscan shells are environmentally, economically and evolutionarily important [1]. The hard multi-functional external shells of molluscs are often attributed to the evolutionary success of this group. Shells are exquisitely preserved in the fossil record and their expansive extant adaptive diversity of form, as well as remarkable phenotypic plasticity, provides a powerful system to study morphological evolution [2]. In the last decade, deciphering the molecular mechanisms that control shell secretion has received particular focus [3]. A range of gastropod [4,5], cephalopod [6,7] and bivalve [8,9] shells have been subject to proteomic sequencing, coupled to a transcriptomic investigation of the shell-secreting mantle tissue [10,11], resulting in large lists of candidate shell-forming genes and proteins. Comparative methods have found that despite the ‘deep’ homology of molluscan shells and shell plates [12], the molecular mechanisms that control biomineralization in molluscs—particularly the downstream effectors such as the shell matrix proteins—are extraordinarily diverse [13]. Features such as repeat low-complexity domains, domain shuffling and co-option [14], gene family expansions and subsequent subfunctionalization [15] drive much of the observed molecular diversity. A handful of core protein domains, however, are essential for all molluscan biomineralization, regardless of morphologies, microstructures or polymorphs (carbonic anhydrase, chitin-binding, VWA and tyrosinase domains [8]). Presumably due to the likelihood of fossilization, ease of study and ability to collect sufficiently large samples, there is an overwhelming bias towards adult shell research, particularly in proteomic and transcriptomic studies. In developmental studies, some candidate gene approaches have been successfully identified [16–19], but comparatively little is known about the molecular processes that pattern and generate the embryonic shell field, larval mantle and early shell secretion.

Phenotypically, larval mollusc shells are more conserved than adult shells in terms of morphology, crystal polymorph and microstructure; they are unsculptured simplified forms composed of an organic outer layer and aragonite mineralized layer [20]. More generally, developmental processes are highly conserved and under strong selective constraints [21,22] and so, if there are deeply homologous molecular mechanisms directing the production of the molluscan shell, we propose they are more likely present during developmental stages. Although limited in number and taxonomic coverage, studies on the molecular control of shell development have uncovered intriguing insights. Recent work in pteriomorphian bivalves (oysters and mussels) compared the proteomes of larval shells to those of adults in the same species and showed that the repertoire of extracellular matrix proteins in the larval shell is almost completely different to that of the adult shell [23,24]. Only the handful of core protein domains, which have been previously described as the core ‘tool-kit’ for molluscan biomineralization, were shared between the two life-history stages (carbonic anhydrase, chitin-binding and VWA). To date, intraspecific comparisons of the molecular mechanisms governing larval versus adult shell production are restricted to one bivalve infraclass (Pteriomorphia, e.g. oysters and mussels). These studies exclusively used proteomics in a qualitative presence versus absence approach, with non-replicated estimates of transcript abundance through ontogeny. They were the first studies to reveal the striking difference between larval and adult shells at the molecular level and they focus solely on the post-translationally modified downstream effectors, i.e. the shell matrix proteins. Much less is known about the transcriptional mechanisms that regulate early shell development, in comparison with the adult shell of the same species.

Here we studied shell development using imaging and quantitative transcriptomics in a member of the Heteroconchia infraclass, the Antarctic clam, *Laternula elliptica*. Heteroconchia split from the rest of the Autobranchia in the Cambrian Period, around 500 Ma [25], and so comparisons within the Autobranchia but between Heteroconchia and Pteriomorphia can shed light on features that are deeply conserved over 500 million years of evolutionary time within the bivalves, and likely ancestral to the Autobranchania.

## 2. Results

### 2.1. Morphological mantle and shell landmarks through development in *Laternula elliptica*

**Figure 1.**
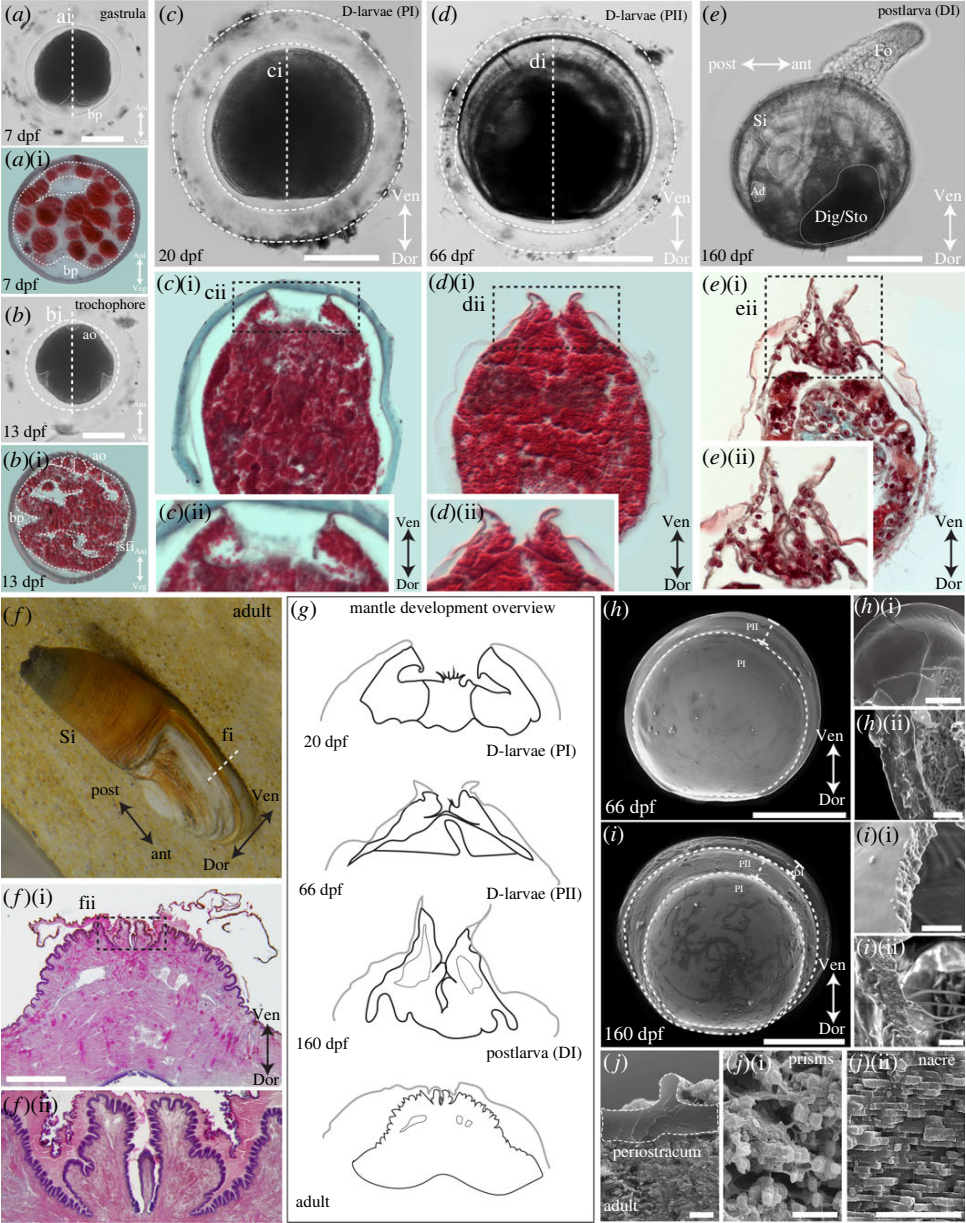
Shell and mantle development in *Laternula elliptica* characterized using live imaging, histology and SEM. (*a*-*a*i) Invagination of the blastopore (bp) during gastrulation 7 days post fertilization (dpf) forming the archenteron prior to shell field induction (encapsulated, scale bar 60 µm). (*b*-*b*i) Early trochophore 13 dpf apical organ (ao) and shell field invagination (sfi) (encapsulated, scale bar 60 µm). (*c*-*c*ii) Early D-stage larvae 20 dpf, first prodissoconch I organic shell (PI) secreted by larval mantle that is unfolded (*c*i*,*ii), reduced ciliary velum resorbing (*c*ii) (encapsulated, scale bar 60 µm). (*d*-*d*ii) Late D-stage larvae 66 dpf with prodissoconch II (PII) that is mineralized, mantle folds appear and fuse to form the early fused inner mantle fold (capsule disintegrating, scale bar 60 µm). (*e*-*e*ii) Hatchling postlarva 166 dpf with mineralized dissoconch (DI) secreted by one cell thick-folded mantle (*e*ii), siphon (Si), adductor muscle attachment (Ad) and digestive gland/stomach (Dig/Sto) are visible through the transparent shell, foot (fo) active (scale bar 100 µm). (*f*-*f*ii) Adult mantle with outer folds and fused inner fold, periostracal grooves secreting two-layered periostracum, scale bar 2 mm image f of adult animal has background filled in for illustrative purposes. (*g*) Schematized overview of mantle morphogenesis, mantle tissue black, periostracum grey. At late D-larvae stage PII (66 dpf), mineralization coincides with the formation of folds in larval mantle edge. (*h*-*h*ii) SEM of late D-larval shell 66 dpf showing delineated PI and PII shells, polygonal mottling on the dorsal surface suggestive of the calcification nucleation points, (*h*i & *h*ii) show the shell is brittle and hence mineralized, (*h*) scale bar 100 µm, (*h*i) scale bar 35 µm and (*h*ii) scale bar 10 µm. (*i*-*i*ii) SEM of postlarval shell 160 dpf DI delineated from PI and PII shells, arenophilic secretions (which are absent in adult shells as per [26]) and intraperiostracal spikes present. (*i*) scale bar 100 µm, (*i*i) scale bar 5 µm and (*i*ii) scale bar 3 µm. (*j*-*j*ii) SEM cross-section of adult shell microstructure, a relatively think periostracum (*j*), an outer layer of aragonitic granular prisms (*j*i) and an inner layer of aragonitic nacreous sheets (*j*ii), scale bars 5 µm.

In order to phenotypically characterize the development of the shell field, larval mantle and shell in *L. elliptica*, a combination of live light imaging, histological staining and scanning electron microscopy (SEM), was used (figure 1). Phenotypic characterizations allowed us to assign key mantle and shell landmarks to each of the development stages studied, such as mantle fold appearance, organic versus mineralized shell, prodissoconch I (PI), prodissoconch II (PII) and dissoconch I (DI) shells, to use as a framework to decipher the transcriptional regulation of shell secretion.

### 2.2. Transcriptional characterization of shell development in *Laternula elliptica*

**Figure 2.**
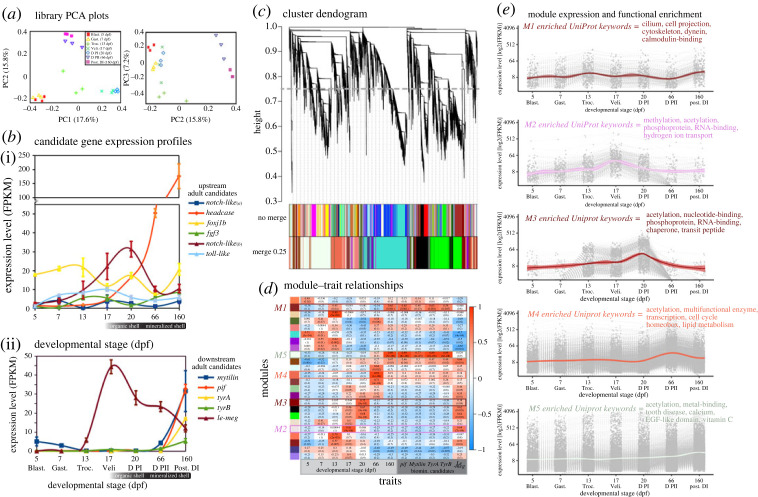
Candidate gene and trait-based approach to decipher transcriptional regulation of mantle and shell development in *Laternula elliptica* using bulk RNA-Seq. (*a*) PCA plots (PC1–3) of each RNA-seq library clustering by developmental stage (*n* = 3 libraries per stage). (*b*) Average expression FPKM, mean average ± s.e., *n* = 3, at each stage of the shell development of candidate upstream regulatory and downstream effector genes. (*c*) Dendrogram obtained by average linkage hierarchical clustering using the WGCNA R package, module assignment determined by the Dynamic Tree Cut after merging at 75% cut-off. (*d*) Correlation of eigengene modules to shell development traits (dpf and expression of candidate downstream adult biomineralization genes). Each row corresponds to an eigengene module and each column a trait, colour-coded by direction and degree of the correlation (Pearson R, red = positive correlation; blue = negative correlation). Five eigengene modules are highlighted (M1–M5) as they significantly positively correlate to shell development traits of interest. (*e*) All transcripts in each eigengene module of interest (M1–M5) extracted and mean average FPKM plotted over developmental time (*n* = 3, shaded ±95% confidence interval, method = loess). Selected biologically interesting significantly enriched Uniprot keywords highlighted (calculated using String-DB, electronic supplementary material).

First, a candidate gene approach was used to ask if the larval shell is built using the same protein-coding genes as the adult shell. The expression of six genes encoding upstream regulatory proteins such as transcription factors and signalling molecules, and five downstream effector genes encoding extracellular matrix proteins and biomineralisation enzymes, was quantified over developmental time. All candidates had previously been characterized in adult shell secretion and repair in this species (via transcriptomics, computational gene network predictions, shell proteomics, qPCR and *in situ* hybridizations [9,27,28]). We found that all the upstream regulatory candidates were expressed during development, either increasing over time, or oscillating between stages (figure 2*b*i). Only one of the downstream candidates, *le-meg,* was expressed during early shell development, the remaining four (*mytilin, pif, tyrA* and *tyrB*) were expressed only in the latest postlarva stage (figure 2*b*ii).

Next, genes with expression profiles indicating likely involvement in regulating shell development were screened. A trait-based approach, using weighted correlation network analysis (WGCNA), was used to cluster expression profiles into modules (figure 2*c*). Module-trait relationships were then calculated to identify sets of genes (eigengenes) whose expression significantly correlated to the previously identified mantle and shell development landmarks. Five eigengene modules (M1–5) were extracted based on their significant positive correlation to traits of interest (*p* < 0.001, *R* > 0.65, figure 2*d–e*). M1 contained 352 genes and was significantly positively correlated to the postlarva stage (DI, mineralized, intraperiostracal spikes and arenophilic secretions) and the average expression profile of two downstream adult candidate shell genes (*pif* and *tyrA*). M1 was enriched in genes related to the cytoskeleton, protein transport (dynein) and calmodulin-binding (genes of interest, including conserved developmental genes, including: *arm*, *ltbp4*, *foxj1*, *vwa3a*, *iqcg* and *camk4*). M2 contained 337 genes whose expression profile significantly positively correlated to the veliger stage 17 dpf (first organic PI initially deposited) and the average expression profile of one downstream adult candidate gene (*le-meg*). M2 was enriched in genes related to methylation and hydrogen ion transport (genes of interest, including conserved developmental genes, including: *alpl*, *chs2*, *sbp1* and *h2b*). M3 contained 647 genes and was significantly positively correlated to early D-larvae (PI) stage 20 dpf (first organic shell) but none of the adult candidate shell genes. M3 was enriched in genes related to protein folding and protein transport (genes of interest, including conserved developmental genes, including: *hsp70*, *cpn601*, *hsp90*, *btf3* and *ccb23*). M4 contained 2875 genes whose expression significantly positively correlated to late D-larvae (PII) stage 66 dpf (mineralized shell) but none of the adult candidate shell genes. M4 was enriched in genes related to proliferation/growth, hox code transcription factors and multi-functional enzymes (genes of interest, including conserved developmental genes, including: *a-somp*, *fgf1*, *abd-a*, *abd-b*, *nkx2.6* and *mox1*). M5 contained 6369 genes significantly positively correlated to postlarva stage (DI) 160 dpf and the average expression profile of four of the adult biomineralization candidate genes (*pif, mytilin, tyrA* and *tyrB*). M5 was enriched in genes related to calcification, egf-like domains and vitamin C (genes of interest, including conserved developmental genes, including: *bmp2*, *bmp3*, *bmp7*, *fgfl-1*, *ca*, *fz4*, *notch1*, *wnt4*, *wnt2b*, *tyr2* and *perl*). Modules 2–4 contained genes whose expression dynamics suggest they are involved in the regulation of early larval shell, whereas M1 and M5 are both correlated to the downstream adult shell genes and show an increase in expression only in the latest stage, the postlarva.

**Figure 3.**
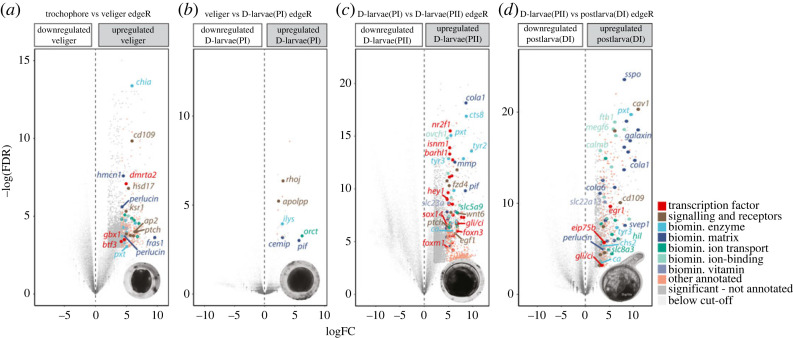
Unbiased pairwise differential expression approach to decipher transcriptional regulation of shell development in *Laternula elliptica*. Differential expression was determined using edgeR with a negative binomial additive general linear model and quasi-likelihood *F*-test, corrected for multiple testing using the Benjamin–Hochberg method to control the FDR. Upregulated genes at each stage screened for statistical significance (FDR < 0.05), magnitude (log_2_FC > 2) and functional categories related to shell development, as per colour-coded key. Specific genes of biological interest are labelled. (*a*) Upregulated genes at initial organic shell stage, veliger 17 dpf. (*b*) Upregulated genes at early D-larvae (PI) stage 20 dpf with full organic PI shell. (*c*) Upregulated genes at late D-larvae (PII) stage 66 dpf when larval shell first begins to be mineralized and folds established in the larval mantle. (*d*) Upregulated genes in hatchling postlarva (DI) stage 160 dpf.

A second unbiased statistical approach was used to identify genes that were upregulated at each consecutive stage of shell development. Significantly upregulated genes were screened for functional categories relating to shell secretion (figure 3), extracted and plotted over time in clustered heatplots (electronic supplementary material, figure S1). Genes upregulated at the D-larvae (PII) stage included a striking number of transcription factors and signalling molecules, such as components of the HH and WNT signalling pathways (*gli*, *ptch*, *fzd4* and *wnt6*). The stage that had the fewest upregulated genes was D-larvae (PI) with 25 genes upregulated including genes involved in signalling (*rhoj* and *apolpp*), ion transport (*orct*) and the extracellular shell matrix (*pif*) (electronic supplementary material). Many of the screened biomineralization genes had a stage-specific expression pattern. For example, genes that were highly upregulated in the postlarva stage (when the downstream adult shell candidates are also highly expressed) were expressed at very low levels through all the earlier stages of shell development. Some of the stage-specific expression patterns were explained by ontogenetic partitioning of isoforms and/or paralogues (indicated with arrows, electronic supplementary material, figure S1), for example, we found an isoform of *ptch3* that was expressed only in early shell development (significantly upregulated in the veliger stage) and a different isoform only expressed late in shell development (significantly upregulated in D-larvae PII stage).

**Figure 4.**
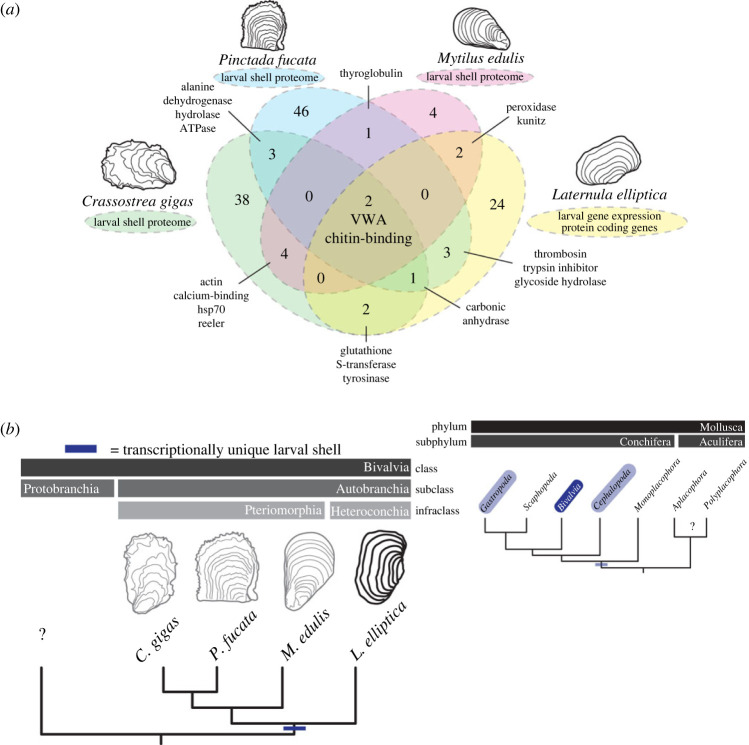
Development-specific downstream effectors control larval shell production in autobranchians, and more broadly across conchiferans. (*a*) Larval downstream effectors such as shell matrix proteins and biomineralization enzymes were translated to amino acid sequences and surveyed for known domains. Protein domains were compared against larval shell proteomes to search for conserved versus lineage-specific domains using OrthoVenn2. (*b*) A development-specific larval shell proteome (versus adult shell proteome) in three species of Pteriomorhpia and, presented here, transcriptionally unique, development-specific downstream shell genes in a representative from Heteroconchia points to an ancestral condition to the Autobranchia subclass. Development-specific shell genes and isoforms have also been reported in gastropods. Taken together with many conchiferan molluscs retaining a larval shell but secondarily losing, or reducing, an adult shell lead us to hypothesize that a transcriptionally unique larval shell is likely a deeply conserved trait to the Conchifera (no known developmental data for Aculifera or Protobranchia).

The signalling components upregulated during the shell development stages include HH and WNT pathways (figure 4*a,b*). To further explore the possible role of these signalling pathways in shell development we extracted genes relating to the canonical transduction of the HH and WNT pathways and plotted them over developmental time (electronic supplementary material, figure S2). Most of the HH and WNT signalling genes were expressed prior to the secretion of the larval shell, followed by very low levels during early shell development stages—veliger and D larvae (PI) stages. One exception was a *ptch* isoform, which was upregulated again at the late D (PII) stage, coinciding with the beginning of larval mantle folding and onset of mineralization.

### 2.3. Interspecies comparison of the molecular control of larval shell development

To test if larval shell matrix proteins are more conserved than those of adults, genes identified as likely larval shell downstream effectors in *L. elliptica* were compared with the three currently publicly available larval shell proteomes (*Crassostrea gigas*, *Pinctada fucata* and *Mytilus edulis*). Protein domains were compared across the four species to search for conserved versus lineage-specific domains, as per Arivalagan *et al*. [8]. Only two protein domains were present in all four species larval shell datasets, VWA and chitin-binding, carbonic anhydrase was present in three out of four species (figure 4*a*).

## 3. Discussion

We have quantified the ontogenetic expression dynamics of genes putatively involved in shell development for the first time in a heteroconchian bivalve, the Antarctic clam *L. elliptica.* These genes can be broadly categorized into two groups: downstream effectors that code for proteins involved in the extracellular shell matrix, enzymes and ion transporters (such as peroxidases, chitinases, matrix metalloproteinases, galaxins, ion and vitamin transporters) and upstream regulatory genes that code for transcription factors and signalling components (such as WNT and HH signalling components, hox, fox and sox transcription factors). Owing to a lack of genetically tractable model systems to conduct gene function experiments in molluscs (and more broadly spiralians), data on the gene regulatory networks that drive processes such as shell development and biomineralization in this group are sparse, either focusing on two or three candidates [18,19,29–32], or using only computational methods [27]. Previous work has implicated BMP signalling in the regulation of molluscan shell secretion using *in situ* expression data and perturbations such as RNAi, DMH1 (a BMP inhibitor) or addition of BMP protein. Results have been conflicting; work in bivalves suggests BMP signalling could play a role in controlling adult shell secretion [33] and shell development [18,19]. More recent experiments in the gastropod *Crepidula fornicata*, however, found that when BMP signalling was disrupted during development, there was no observed effect on shell phenotype [34], and single-cell RNA-seq data from the trochophore stage of the heteroconchian bivalve *Dreissena rostriformis* suggests no BMP gene expression in shell field cells [35]. The WGCNA analysis presented here found BMP pathway components were correlated only to the postlarva stage and the upregulation of the downstream adult shell candidates, but no BMP components were found using the differential expression approach. Taken together with previous reports in other groups, we hypothesize that BMP signalling may not be involved in early shell development in heteroconchian bivalves, but could play a role in controlling the growth and maintenance of shell secretion at later stages. In addition, we hypothesize that, similar to the story emerging on the diverse roles BMP signalling plays in patterning the nervous system across the Bilateria [36], the BMP pathway is likely functioning in diverse roles in shell development across molluscs.

The transcriptomic profiling data presented here suggest that HH and WNT signalling pathways are involved in shell development. These signalling pathways have well-characterized roles in animal dorsal–ventral (WNT) and anterior–posterior patterning (HH and WNT), and the outgrowth of appendages in vertebrates and arthropods, as well as osteoblast differentiation and digit patterning in vertebrate endoskeletons [37–40]. Previous work has linked WNT signalling genes to biomineralization in bivalves [41], and most recently, WNT and HH signalling have been shown to pattern cephalopod arm development [42]. If cephalopod limbs evolved by parallel activation of a gene regulatory network for appendage development that was present in the bilaterian common ancestor [42], our data also suggest that this genetic program for appendage development may have been co-opted for shell development. More data are required on the spatial expression and function of HH and WNT genes in relation to mollusc shell development, and future work in species more suited to such methods should prioritize these studies.

Recent larval shell proteomes of three pteriomorphian bivalve species report that a development-specific repertoire of downstream effector proteins are found in the extracellular matrix of the larval shell when compared to the adult shell [23,24]. Here, we asked if this pattern holds true in a heteroconchian representative and aimed to understand the transcriptional regulation of larval shell deposition versus that of adults. We find that previously characterized downstream adult shell genes are largely not involved in larval shell secretion (except for *le-meg*), neither the first organic stage nor early mineralized stages, but that the upstream regulatory genes, such as signalling molecules and transcription factors, are more conserved between life-history stages. Our data suggest that global transcriptional mechanisms underpinning the unique larval shell proteins include ontogenetic partitioning of isoforms and paralogues, in addition to some potential *bona fide* unique larval shell genes candidates (such as *btf3*, *pap18*, *chid1*, *cemip* and *nsf*). We hypothesize that, similar to other developmental systems, such as *svb*-driven gene regulatory networks in the formation of diverse actin-rich projections in arthropods [43], conserved gene regulatory networks drive shell deposition in larval and adult life-history stages, but the downstream effectors activated by the network depends on local factors in different life-history and cellular contexts. As is the case with many systems studying morphological evolution, and as Smith *et al*. [43] discuss, questions regarding the connection between gene regulatory networks and downstream effectors, the generation of temporal and local contexts and how different downstream effectors exert influences on phenotypic outcomes, remain unanswered.

Terminal downstream effectors in adult shells, such as extracellular shell matrix proteins, are rapidly evolving, lineage-specific and surprisingly diverse, with just four domains thought to be critical for all molluscan biomineralization (carbonic anhydrase, chitin-binding, VWA and tyrosinase [8]). To test if this pattern holds true for early life-history stages where, phenotypically at least, shells are more conserved between species, we carried out comparative domain analysis between heterochoncian and pteriomorphian bivalves. Similar to the pattern in adult life-history stages for these groups, we find only two domains shared between the four species (chitin-binding and VWA domains), despite the similarity in shell phenotype across species at this stage. In our analysis, we compared data generated by different groups using different methods, in addition, the data we present here for *L. elliptica* is a prediction of biomineralization effectors, and hence, it is likely that the number of conserved domains are underestimated. For example, it is perhaps unlikely that carbonic anhydrase is truly absent in larval mussel shells as reported by Carini *et al*. [23], but rather it was technologically difficult to detect all proteins present in larval shell with the small input material that is available from developmental stages.

For the first time, we have compared the transcriptional regulation of shell secretion at different life-history stages of a heteroconchian bivalve. Studying a heterconchian representative, and comparing it to data available for pteromorphians allows us to hypothesize that a development-specific transcriptionally unique larval shell is likely an ancestral feature of autobranchians, or perhaps even more broadly to conchiferans (figure 4*b*). Ontogenetic partitioning of some specific shell gene isoforms has been reported in gastropods, as well as alternative splicing to generate diverse shell matrix proteins from a single genomic loci (*Halitosis asinina* and *Lymnaea stagnalis* [4,13]). In addition, some conchiferan molluscs have a larval shell but have secondarily lost, or reduced, an adult shell [17]. There is a huge diversity of form and function between different life-history stages in molluscs. Taken together, these findings lead us to hypothesize that a development-specific transcriptionally unique larval shell may be a deeply conserved trait to the Conchifera, but more data from diverse taxa, especially the Acuilifera, are needed to resolve questions on the evolution of biomineralization in the molluscs.

## 4. Methods

### 4.1. Animal husbandry, spawning and developmental staging

Embryos were obtained from an adult broodstock of sexually mature *L. elliptica* individuals held at the British Antarctic Survey, Cambridge, UK and divided into three independent closed-system 1 l tanks. Embryos were maintained at 0°C ± 0.5°C, aerated with an airstone with 50% water changes every 2 days using autoclaved seawater. Embryos were staged as per Peck *et al*. [44].

### 4.2. Fixation, histology and imaging

For histology and SEM, embryos were fixed in 500 µl of 2.5% glutaraldehyde in phosphate-buffered saline (PBS) at room temperature for 30 min, rinsed twice in PBS with 0.1% tween, dehydrated into 100% ethanol and stored at 4°C.

For histology, embryos were cleared in Histosol (National Diagnostics, 3 × 20 min, room temperature), transitioned through 1 : 1 Histosol:molten paraffin (2 × 30 min, 60°C) then pure molten paraffin (RA Lamb Wax – Fisher Scientific, 60°C overnight). Five changes of molten paraffin (each greater than 1 h) were conducted before embedding in a Peel-A-Way embedding mould (Sigma). Wax blocks were serially sectioned at 8 µm on a Leica RM2125 rotary microtome. Sections were stained with a modified Masson's trichrome stain as per Witten and Hall [45]. All histology was conducted on a minimum of three individuals per stage. Sections were imaged on a Zeiss Axioscope A1 with Zen software (at University of Cambridge).

For SEM of the shell late D (66 dpf) and postlarval (160 dpf) stages, larvae were transferred from ethanol to electron microscope stubs, attached using carbon adhesive discs, carbon coated and examined using a QEMSCAN 650F (at University of Cambridge) at accelerating voltages of 10 kV.

Each stage was also live imaged on an upright compound light microscope fitted with a camera (Olympus BX50 microscope fitted with Olympus PM-C35 camera using Olympus U-CMAD-2 software, at the British Antarctic Survey) by mounting in seawater onto a glass slide, under a coverslip on small clay feet. All live imaging was conducted on a minimum of three individuals per stage.

Images were adjusted for contrast and brightness, cropped, rotated and flipped. All plates were constructed in Adobe Illustrator software.

### 4.3. Sampling, RNA extraction and sequencing

Triplicate RNA-Seq samples were collected for each developmental stage, one from each independent tank. For each sample, exactly 200-staged-matched embryos were snap frozen in a 70% ethanol dry ice slurry and stored at –80°C. Total RNA was extracted from each sample as per manufacturer's recommendations (Relia Miniprep kit, Promega) and tested for quality and quantity using Nanodrop and Agilent Tapestation. All samples had an RNA integrity number of over 7. Libraries were prepared by the DNA sequencing facility in the Biochemistry Department at the University of Cambridge (TruSeq Stranded mRNA, Illumina) and sequenced on an Illumina NextSeq500 generating over 300 million 150 bp stranded paired-end reads. All raw data are publicly available from NCBI SRA accession number: PRJNA803976.

### 4.4. Bioinformatics and statistical analyses

Raw reads (total 309 593 642) were cleaned using ea-utils tool v1.1.2 fastq-mcf (quality –q 30, and length –l100), after cleaning 296 480 254 reads remained. Prior to *de novo* assembly, reads were normalized using the Trinity v.2.2.0 utility script (insilico_read_normalization.pl –max_cov 50), leaving 64 355 068 reads for assembly. Clean, normalized reads were assembled using Trinity v.2.2.0 with default parameters [46,47]. Assembly quality was assessed using Trinity utilities and the gVolante webserver tool (electronic supplementary material, table S1). Transcript abundance was estimated by alignment-based quantification using Trinity v.2.2.0 utilities. Transcripts from each cleaned library were aligned to the transcriptome using bowtie2 with default parameters and transcript abundance estimates were calculated using RNA-seq by expectation-maximization. Raw counts and trimmed mean of M-values (TMM) normalized Fragments Per Kilobase Of Exon Per Million Fragments Mapped (FPKM) matrices were generated (available to download from BioStudies accession S-BSST926).

WGCNA was used to find clusters, termed modules, of genes with highly correlated expression across all libraries. Each cluster was then correlated to external traits of interest [48]. The raw counts matrix was loaded in R and EdgeR [49] functions were used to remove lowly expressed transcripts (keep transcripts with cpm greater than 5 in greater than or equal to four libraries) leaving 37 000 transcripts for WGCNA. TMM-FPKM values were used to calculate a gene dissimilarity matrix (adjacency = softpower 16 and signed, TOMsimilarity = signed) and hierarchical clustering was performed (method = average). Modules were determined using the cutreeDynamic function with a minimum gene membership threshold of 30 and dynamic tree cut-off of 25. Modules were correlated to external traits (days post fertilization or average candidate gene expression values). Modules that were significantly correlated to traits of interest were extracted and all transcripts were putatively annotated based on sequence similarity searched using blastx against Uniprot (http://www.uniprot.org/) and tested for functional enrichment. Scripts, module genes identities and annotation results are available [50], full details in the electronic supplementary material information.

Pairwise differential gene expression tests were performed to find transcripts that were upregulated at each stage of shell development (compared to the previous stage). Using the EdgeR package, a negative binomial additive general linear model with a quasi-likelihood *F*-test was performed and *p*-values were adjusted for multiple testing using the Benjamini-Hochberg method to control the false discovery rate, cut-offs for statistical significance (FDR ≤ 0.05) and magnitude were used (log_2_FC > 2). See electronic supplementary material information for access to analysis scripts. Upregulated transcripts were putatively annotated based on sequence similarity searched using blastx against Uniprot (http://www.uniprot.org/) and screened for functional categories relating to gene regulation and shell secretion. Differential expression results and annotations are available for download (electronic supplementary material information).

All upregulated genes that were functionally categorized as downstream effectors, such as shell matrix proteins and biomineralization enzymes, were translated to amino acid sequences and surveyed for known domains. Protein domains were then compared against published larval shell proteomes for oyster [24] and mussel [23] species to search for conserved versus lineage-specific domains as per Arivalagan *et al*. [8], using OrthoVenn2.

## Data Availability

Raw read data generated in this publication are freely available at NCBI SRA with the following accession: PRJNA803976; transcriptome assembly, data matrices and candidate gene files are available from BioStudies (accession S-BSST926). All analysis scripts are available from: https://github.com/SleightLab/Lelliptica_bulkRNAseq_analysis. All analysis results tables are available from the UK Polar Data Centre, Sleight *et al*. [50]: https://doi.org/10.5285/6CD12DE1-02C7-4F94-86F0-C11E76B86067. The data are provided in the electronic supplementary material [51].
